# Epworth sleepiness scale is associated with hypothyroidism in male patients with obstructive sleep apnea

**DOI:** 10.3389/fendo.2022.1010646

**Published:** 2022-11-16

**Authors:** Le Wang, Xiaoyan Fang, Chong Xu, Na Pan, Yan Wang, Tuai Xue, Mingchu Zhang, Jie Cao, Jing Zhang

**Affiliations:** Department of Respiratory and Critical Care Medicine, Tianjin Medical University General Hospital, Tianjin, China

**Keywords:** epworth sleepiness scale, obstructive sleep apnea, overt hypothyroidism, subclinical hypothyroidism, thyroid

## Abstract

**Background:**

Hypothyroidism could cause obstructive sleep apnea (OSA), however, the specific association of them remained unclear. This cross-sectional study aimed to determine the prevalence of hypothyroidism among patients with OSA, and the characteristics and predictors of hypothyroidism associated with OSA.

**Methods:**

A total of 573 patients with OSA were included in the study. Serum levels of thyroid stimulating hormone (TSH), free triiodothyronine (FT3) and free thyroxine (FT4) were measured in all participants. Univariate and binary logistic regression analysis were performed to assess the association of OSA with hypothyroidism while controlling for potential confounders. Receiver operating characteristic (ROC) curve analysis was performed to evaluate the OSA effect in the distinction between euthyroid and hypothyroidism.

**Results:**

The prevalence of hypothyroidism was 6.75%、5.12%、10.38% in the total, men, and women cohort, respectively, and the prevalence rate in women OSA patients was significantly higher than that in men OSA patients (P=0.018). The men OSA patients with hypothyroidism had a higher Epworth sleepiness scale (ESS) than women OSA patients with hypothyroidism (P=0.022). Additionally, the ESS was significantly higher in men OSA patients with hypothyroidism than those with euthyroid (P=0.042), while women OSA patients had no such difference (P=0.822). In men patients with OSA, ROC curve analyses revealed that the risk of hypothyroidism increased in accordance with increasing ESS after adjustment for potential confounders, and the optimal cutoff value was 10 score. Higher ESS category was significantly associated with a higher risk of prevalent hypothyroidism in men patients with OSA [odds ratio (OR) = 4.898 for ESS≥10 relative to ESS <10, 95% confidence interval (CI) 1.628-14.731, P = 0.005].

**Conclusions:**

The prevalence of hypothyroidism in OSA patients was relatively higher, especially in women OSA patients. ESS was significantly and positively associated with hypothyroidism in men patients with OSA, suggesting that ESS may have a potential role in identification and diagnosis of men OSA patients complicated with hypothyroidism.

## Introduction

Obstructive sleep apnea (OSA) is a kind of common sleep-disordered breathing with significant characteristic of snoring, witnessed apnea, excessive daytime sleepiness and fatigue. It was estimated that the overall population prevalence of OSA ranged from 9% to 38% (1), and even exceeded 50% in some countries (2). Extensive research has shown that OSA has a far-reaching health impact due to intermittent hypoxia and hypercapnia resulting from recurrent, partial or complete collapse of upper airway, leading to increased overall mortality and morbidity rates (3, 4).

OSA is a syndrome with metabolic and endocrine complications. It was noted that OSA is related to thyroid diseases, especially hypothyroidism (5).The prevalence of OSA was estimated to be 25%-50% in patients with hypothyroidism (6). Although the specific pathophysiologic mechanism remains relatively elusive, the increased incidence of OSA in patients with hypothyroidism may be related to obesity, macroglossia, increased thyroid size, myxedema of the upper airway, deposition of mucopolysaccharides in upper airway tissues, and decreased ventilation control (7). Patients with OSA and those with hypothyroidism often present similar symptoms, such as apathy, lethargy, fatigue and excessive daytime somnolence (8).The recognition of the association between two disorders is essential because the overlap between two disorders may result in a misdiagnosis or under-recognition of one of them, and treating one disease does not alleviate another (9). However, the majority of sleep medicine research focused on the impact of OSA on cardiovascular and neurologic health (5), only few papers have reported data about OSA and hypothyroidism, and the views remain controversial (10).

Therefore, this cross-sectional study aimed to investigate the prevalence of hypothyroidism in patients with OSA, and explore the characteristics and predictors of hypothyroidism associated with OSA.

## Methods

### Study design

**Figure 1:**
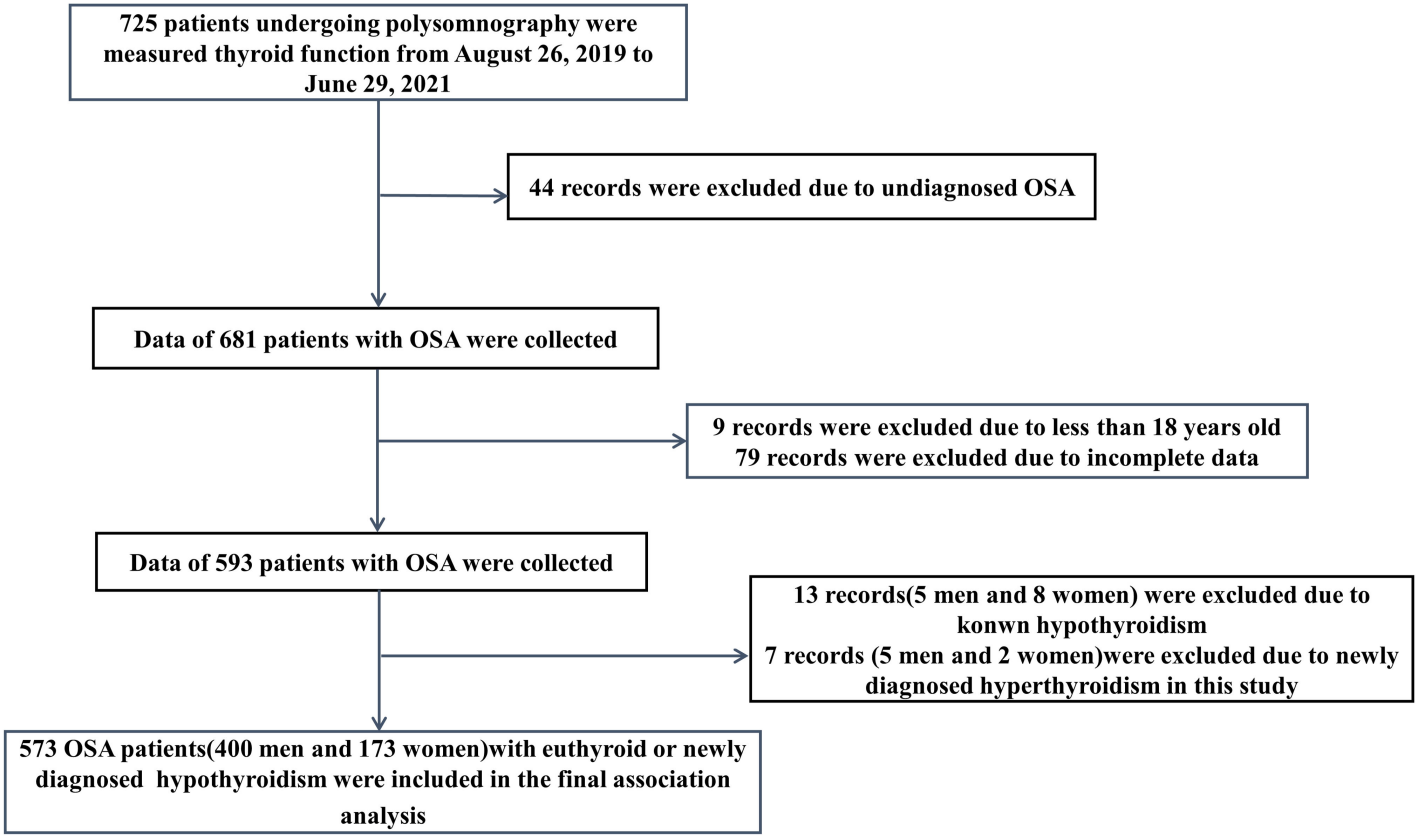
Flow chart of study population.

In this retrospective descriptive study, there were 725 patients undergoing polysomnography (PSG) and measured the thyroid function, including the serum levels of thyroid stimulating hormone (TSH), free triiodothyronine (FT3) and free thyroxine (FT4), from August 26, 2019 to June 29, 2021. The patients diagnosed with OSA according to criteria were included in the study (11). Exclusion criteria were age less than 18 years old, with acute manifestations of any disease and in an unstable clinical state, previous treatment for OSA, current treatment with drugs possibly interfering with thyroid function, with known thyroid disease, with newly diagnosed hyperthyroidism in this study, and incomplete data. The chart of patients flow through the study is summarized in **Figure 1**. Eventually, 573 patients with euthyroid or newly diagnosed hypothyroidism were included in the final association analysis. The study protocol was approved by the Ethics Committee of Tianjin Medical University General Hospital (NO.IRB2022-WZ-101). All procedures performed in the study involving human participants were in accordance with the 1964 Helsinki Declaration and its later amendments. The requirement for informed consent was waived because the patients’ information was extracted from electronic medical records at the sleep center, and the patients’ identities were maintained anonymous.

### Clinical and PSGData

Demographic and anthropometric data, including gender, age, smoking habit, alcohol consumption, medical history, family medical history, height, weight, neck circumference (NC), and waist circumference (WC), were obtained from medical records. The Epworth sleepiness scale (ESS) was used for a subjective assessment of daytime sleepiness. PSG (Alice 5 Diagnostic Sleep System; Philips Respironics, Bend, OR, USA) was performed on all patients during spontaneous sleep under technician control in a single room. Electroencephalography, electrooculography, submental and leg electromyography, electrocardiography, airflow in the mouth and nose, chest and abdominal respiratory efforts, blood oxygen saturation, snoring, and body position parameters were recorded throughout night. Apnea was defined as cessation of oronasal airflow lasting for≥10s and hypopnea was defined as a 30% or greater decrease in oronasal airflow lasting for more than 10s accompanied by arousal in electroencephalogram and/or a≥3% decrease in arterial oxygen saturation relative to the baseline level. The apnea hypopnea index (AHI) was calculated as the total number of apnea and hypopnea episodes per hour. OSA severity was defined by AHI as follows: non-OSA (AHI<5), mild OSA (5≤AHI<15), moderate OSA (15≤AHI<30), and severe OSA (AHI≥30) (11).

### Measurements of thyroid function

5mL fasting venous blood samples were collected at 6:00-7:00 am in the morning after PSG. And the serum levels of TSH, FT3 and FT4 were measured using chemiluminescence method at the clinical laboratory of the Tianjin Medical University General Hospital. ranges for thyroid tests were 0.350-4.940uIU/mL for TSH, 2.43-6.01pmol/L for FT3, and 9.01-19.05pmol/L for FT4. Known thyroid diseases were based on self-reported diagnosis. Euthyroid was defined as serum TSH, FT3, and FT4 levels were within the normal range. The newly diagnosed hypothyroidism included overt hypothyroidism and subclinical hypothyroidism. Overt hypothyroidism was defined as TSH level>4.940 and FT4 level<9.01; subclinical hypothyroidism was defined as TSH level>4.940 accompanied by FT3 and FT4 level within normal range (12). The newly diagnosed hyperthyroidism included overt hyperthyroidism and subclinical hyperthyroidism. Overt hyperthyroidism was defined as TSH level<0.350 accompanied by FT3 level>6.01 and/or FT4>19.05; subclinical hyperthyroidism was defined as TSH level<0.350 in the presence of a normal FT3 and FT4 level (13).

### Statistical analysis

Normal distribution and homogeneity of the variances were evaluated using Kolmogorov-Smirnov test. Continuous variables are shown as mean ± standard deviation (SD) or median with interquartile range (IQR); categorical variables are presented as numbers with frequencies. Differences between the groups were evaluated using Student’s t-tests, Mann-Whitney U tests, chi-squared tests, or Fisher’s exact tests. Univariate and binary logistic regression analysis were used to test the association of OSA with hypothyroidism while controlling for potential confounders. The results are shown as odds ratios (ORs) and 95% confidence intervals (CIs). An analysis of the receiver operating characteristic (ROC) curve and the area under the curve (AUC) was performed to evaluate the OSA effect in the distinction between euthyroid and hypothyroidism. The best cut-off in terms of ROC analysis was identified by the Youden method. A two-sided P-value<0.05 was statistically significant. SPSS 25.0 (SPSS Inc., Chicago, IL, USA) was used for statistical analysis, and GraphPad Prism 8.0 (GraphPad, San Diego, CA, USA) was used to construct figures.

## Results

### Gender differences in OSA patients with euthyroid or hypothyroidism

**Table 1** presents a comparison between the men and women OSA patients with and without hypothyroidism. Euthyroid women OSA patients were heavier than euthyroid men OSA patients, however, euthyroid men OSA patients had a higher NC, rate of smoking and drinking, ESS, AHI, oxygen desaturation index (ODI) and arousal index (ArI), and have a lower minimum percutaneous oxygen saturation (minSpO_2_) and mean percutaneous oxygen saturation (meanSpO_2_), and spent more time with SpO_2_ below 90% compared with euthyroid women (all P<0.001). In the OSA patients with hypothyroidism, there were no significant gender differences except that men patients had a higher alcohol consumption rate and ESS (all P<0.05).

**Table 1:** Gender differences in OSA patients with euthyroid or hypothyroidism.

	Euthyroid (n=546)			Hypothyroidism (n=27)		
	Men (n=384)	Women (n=162)	P	Men (n=16)	Women (n=11)	P
Age, years	43.53±13.00	43.21±15.19	0.814	39.69±14.66	43.09±11.39	0.524
BMI, Kg/m^2^	30.78±6.81	33.99±8.15	<0.001	31.91±7.95	38.39±11.57	0.096
NC, cm	42.71±3.49	39.07±3.38	<0.001	42.63±3.05	40.09±6.24	0.233
WC, cm	107.60±15.70	107.95±16.84	0.823	109.25±17.58	114.36±25.32	0.540
Current smoking, %	211 (54.95)	15 (9.26)	<0.001	7 (43.75)	3 (27.27)	0.488
Current drinking, %	205 (53.39)	13 (8.02)	<0.001	11 (68.75)	1 (9.09)	0.005
Family history of hypothyroidism, %	0	1 (0.62)	0.297	0	0	–
ESS, score	7.00 (4.00,11.00)	5.00 (2.00,8.00)	<0.001	10.00 (7.00,14.00)	6.00 (2.00,10.00)	0.022
AHI,/hr	53.10 (27.20,72.18)	25.10 (13.60,48.90)	<0.001	59.65 (18.50,79.00)	17.40 (10.80,41.30)	0.278
ODI,/hr	44.85 (21.68,68.60)	20.75 (9.28,46.35)	<0.001	57.50 (17.85,78.03)	16.70 (11.00,33.70)	0.217
ArI,/hr	29.35 (16.55,46.38)	16.35 (9.90,26.78)	<0.001	30.65 (17.80,46.15)	12.90 (9.70,61.90)	0.183
minSpO_2_, %	75.00 (63.00,84.00)	81.00 (74.00,88.00)	<0.001	66.00 (59.00,85.00)	81.00 (53.00,87.00)	0.415
meanSpO_2_, %	94.00 (91.00,95.00)	95.00 (93.00,96.00)	<0.001	92.00 (90.00,94.00)	95.00 (91.00,96.00)	0.082
T90, %	8.85 (1.00,29.65)	0.55 (0.00,8.05)	<0.001	25.95 (1.43,36.18)	0.70 (0.06,16.20)	0.108

hypothyroidism included newly diagnosed overt hypothyroidism and newly diagnosed subclinical hypothyroidism.

BMI, body mass index; NC, neck circumference; WC, waist circumference; ESS, Epworth sleepiness scale; AHI, apnea hypopnea index; ODI, oxygen desaturation index; ArI, arousal index; minSpO_2_, minimum percutaneous oxygen saturation; meanSpO_2_, mean percutaneous oxygen saturation; T90, proportion of cumulative sleep time with SpO_2_ below 90% in total sleep time.

### With-gender comparison between OSA patients with euthyroid and hypothyroidism

**Table 2** shows the clinical and polysomnographic characteristics of the study population grouped according to gender and the presence of hypothyroidism. The prevalence of hypothyroidism was 6.75% (40/593)、5.12% (21/410)、10.38% (19/183) in the total, men, and women cohort, respectively, and the prevalence rate in women patients was significantly higher than that in men patients (P=0.018). There was no significant difference in age, body mass index, NC, WC, smoking and drinking history, family history of hypothyroidism, AHI, ODI, ArI, minSpO_2_, meanSpO_2_, proportion of cumulative sleep time with SpO_2_ below 90% in total sleep time (T90) and the serum FT3 and FT4 levels between OSA patients with hypothyroidism and those with euthyroid, whether in the total cohort or in the men/women cohort (all P>0.05). The serum TSH level among patients with hypothyroidism was statistically higher than those with euthyroid (all P<0.05). The ESS was significantly higher in men OSA patients with hypothyroidism than those with euthyroid (P=0.042), while women OSA patients had no such difference (P=0.822).

**Table 2:** With-gender comparison between OSA patients with euthyroid and hypothyroidism.

	Total (n=573)			Men (n=400)			Women (n=173)		
	Euthyroid	Hypothyroidism	P	Euthyroid	Hypothyroidism	P	Euthyroid	Hypothyroidism	P
Participants,%	546 (95.29)	27 (4.71)	–	384 (96.00)	16 (4.00)		162 (93.64)	11 (6.36)	–
Age, years	43.44±13.67	41.07±13.30	0.381	43.53±13.00	39.69±14.66	0.250	43.21±15.20	43.09±11.39	0.974
BMI, Kg/m^2^	31.73±7.38	34.55±9.92	0.157	30.78±6.81	31.91±7.95	0.519	33.99±8.15	38.39±11.57	0.094
NC, cm	41.63±3.84	41.59±4.68	0.960	42.71±3.49	42.63±3.05	0.924	39.07±3.38	40.09±6.24	0.604
WC, cm	107.71±16.03	111.33±20.77	0.379	107.61±15.70	109.25±17.58	0.683	107.95±16.84	114.36±25.32	0.240
Current smoking, %	226 (41.39)	10 (37.04)	0.654	211 (54.95)	7 (43.75)	0.378	15 (9.26)	3 (27.27)	0.058
Current drinking, %	218 (39.93)	12 (44.44)	0.640	205 (53.39)	11 (68.75)	0.227	13 (8.02)	1 (9.09)	0.900
Family history of hypothyroidism, %	2 (0.37)	0 (0.00)	0.908	1 (0.26)	0 (0.00)	0.960	1 (0.62)	0 (0.00)	0.936
ESS, score	6.00 (3.00, 10.00)	10.00 (4.00,12.00)	0.151	7.00 (4.00,11.00)	10.00 (7.00,14.00)	0.042	5.00 (2.00,8.00)	6.00 (2.00,10.00)	0.822
AHI,/hr	42.55 (20.38,71.40)	41.30 (11.50,78.70)	0.779	53.10 (27.20,72.18)	59.65 (18.50,79.00)	0.615	25.10 (13.60,48.90)	17.40 (10.80,41.30)	0.796
ODI,/hr	36.50 (16.58, 64.68)	33.70 (14.20,77.20)	0.956	44.85 (21.68,68.60)	57.50 (17.85,78.03)	0.490	20.75 (9.28,46.35)	16.70 (11.00,33.70)	0.958
ArI,/hr	23.10 (13.48,43.43)	24.70 (12.80,47.00)	0.808	29.35 (16.55,46.38)	30.65 (17.80,46.15)	0.767	16.35 (9.90,26.78)	12.90 (9.70,61.90)	0.884
minSpO_2_, %	77.00 (65.00, 85.00)	73.00 (58.00,86.00)	0.290	75.00 (63.00,84.00)	66.00 (59.00,85.00)	0.342	81.00 (74.00,88.00)	81.00 (53.00,87.00)	0.357
meanSpO_2_, %	94.00 (92.00, 95.00)	93.00 (90.00,95.00)	0.131	94.00 (91.00,95.00)	92.00 (90.00,94.00)	0.061	95.00 (93.00,96.00)	95.00 (91.00,96.00)	0.508
T90, %	4.75 (0.3, 23.44)	14.60 (0.4, 35.90)	0.520	8.85 (1.00, 29.65)	25.95 (1.43,36.18)	0.250	0.55 (0.00, 8.05)	0.70 (0.06,16.20)	0.713
TSH, uIU/mL	1.87 (1.33, 2.58)	5.73 (5.19,7.38)	<0.001	1.80 (1.27,2.42)	5.42 (5.20,7.36)	<0.001	2.11 (1.47,2.80)	5.78 (5.16,7.38)	<0.001
FT3, pmol/L	4.61 (4.21,5.00)	4.43 (4.07,5.00)	0.453	4.68 (4.30,5.13)	4.81 (4.35,5.28)	0.510	4.35 (4.06,4.73)	4.09 (3.72, 4.48)	0.081
FT4, pmol/L	12.40 (11.61,13.37)	12.28 (11.09,13.46)	0.599	12.37 (11.61,13.33)	13.00 (11.59,13.80)	0.548	12.53 (11.58,13.45)	12.10 (10.90,12.82)	0.109

hypothyroidism included newly diagnosed overt hypothyroidism and newly diagnosed subclinical hypothyroidism.

BMI, body mass index; NC, neck circumference; WC, waist circumference; ESS, Epworth sleepiness scale; AHI, apnea hypopnea index; ODI, oxygen desaturation index; ArI, arousal index; minSpO_2_, minimum percutaneous oxygen saturation; meanSpO_2_, mean percutaneous oxygen saturation; T90, proportion of cumulative sleep time with SpO_2_ below 90% in total sleep time; TSH, thyroid stimulating hormone; FT3, free triiodotironine; FT4, free thyroxine.

### Association of ESS and hypothyroidism in patients with OSA

**Figure 2:**
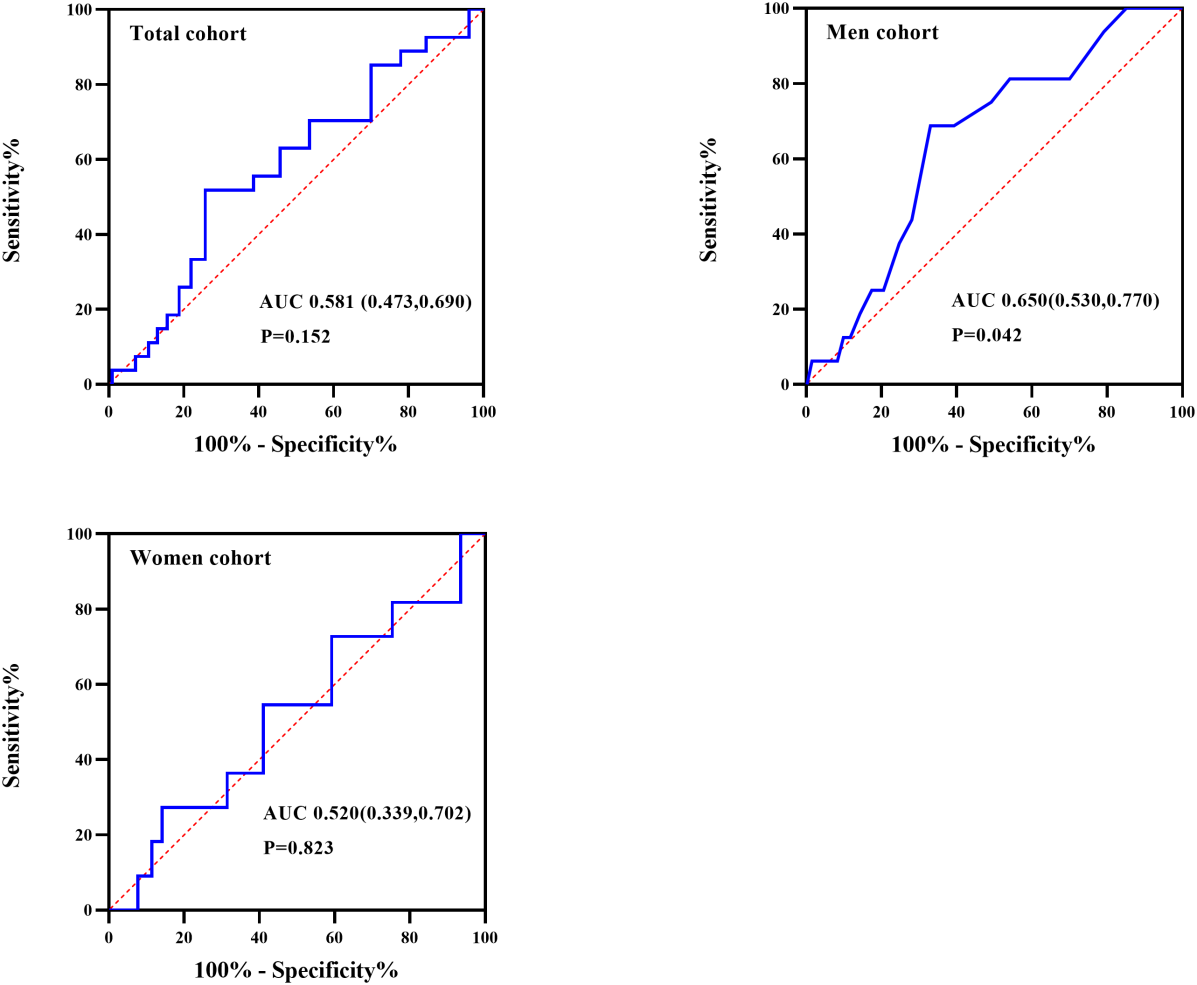
Receiver operating characteristic (ROC) curve analysis of Epworth sleepiness scale (ESS) to recognize hypothyroidism in total, men, and women participants with OSA.

**Figure 3:**
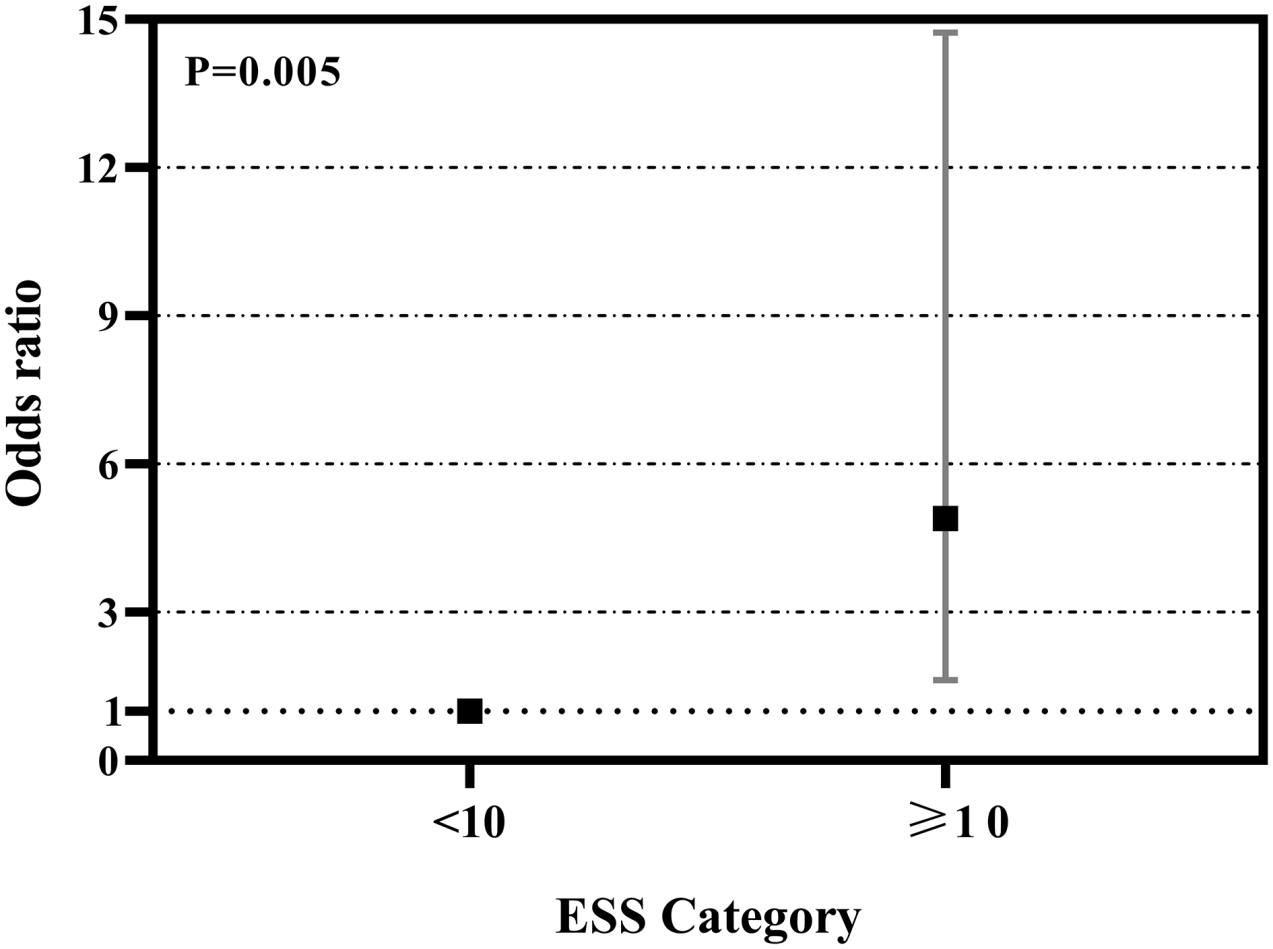
Odds ratios (OR) and 95% confidence interval (CI) of hypothyroidism by ESS category in men patients with OSA. †Adjusted for age, BMI, smoking, drinking, and family history of hypothyroidism.

As showed in **Figure 2**, ROC curve analyzed the ESS for identifying hypothyroidism in patients with OSA. The AUCs of ESS were 0.581(95% CI: 0.473-0.690, P=0.152), 0.650(95% CI: 0.530-0.770, P=0.042) and 0.520(95% CI: 0.339-0.702, P=0.823) for total patients, men patients, and women patients, respectively. The best cut-off to discriminate hypothyroidism in men patients with OSA was ESS ≥ 10, with the sensitivity of 68.8% and the specificity of 69.9%. **Figure 3** summarized the result from binary logistic regression estimating the risk of prevalent hypothyroidism in men patients with OSA based on ESS category after adjusting for age, BMI, smoking habit, alcohol consumption, and family history of hypothyroidism. Higher ESS category was significantly associated with a higher risk of prevalent hypothyroidism in men patients with OSA (OR = 4.898 for ESS≥10 relative to ESS <10, 95% CI 1.628–14.731, P = 0.005).

### Prevalence of hypothyroidism by category of ESS in male patients with OSA

**Figure 4:**
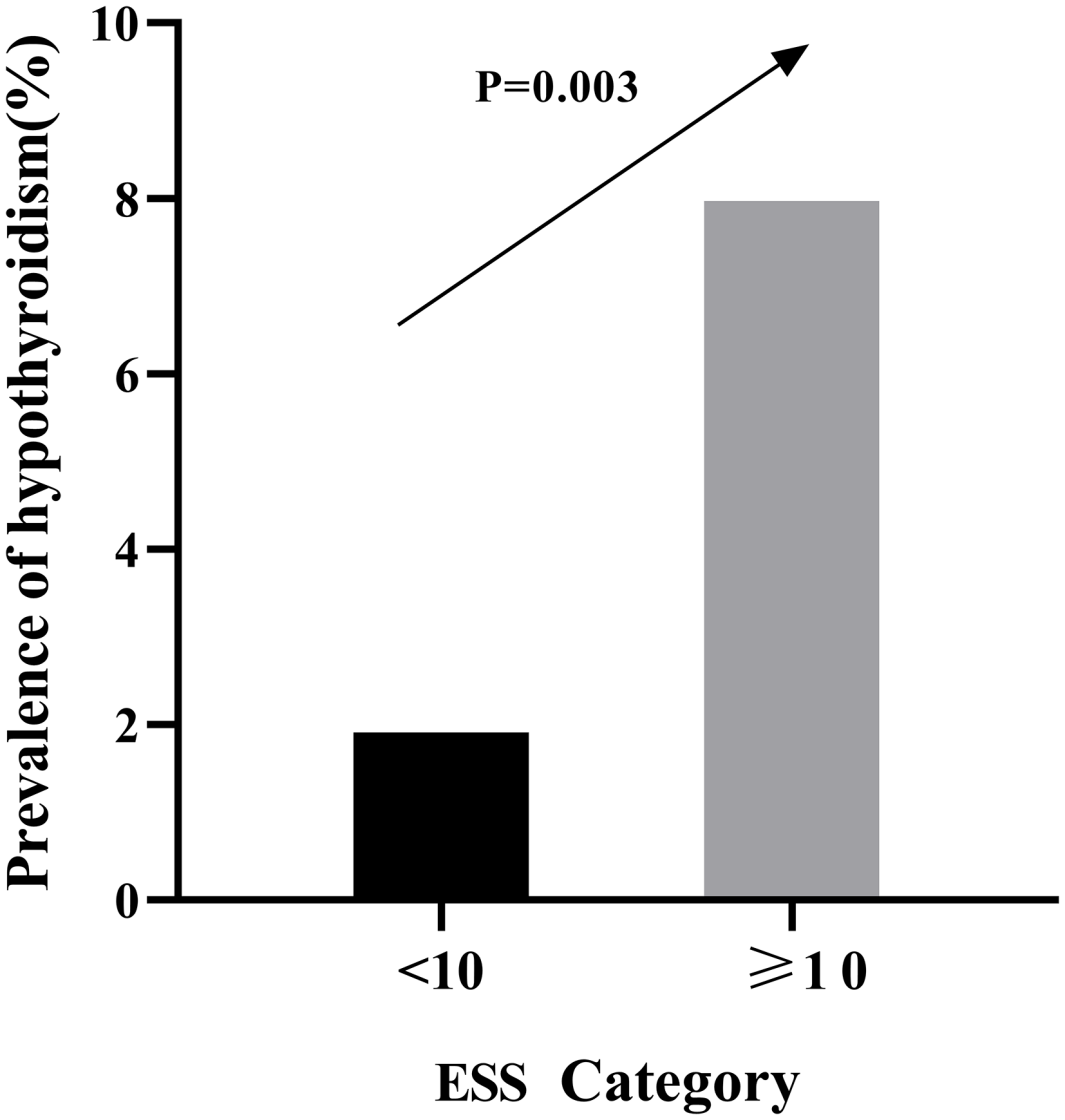
Prevalence of hypothyroidism by category of ESS in men patients with OSA.

The prevalence of hypothyroidism by category of ESS in men patients with OSA was shown in **Figure 4**. The percentages of hypothyroidism increased in accordance with increasing category of ESS (1.91% in ESS<10 relative to 7.97% in ESS≥10, P=0.003).

## Discussion

In this cross-sectional study of 573 OSA patients with no previous diagnosis of thyroid dysfunction, we found a relatively high prevalence of hypothyroidism in patients with OSA, especially in women OSA patients. The ESS was showed to be significantly and positively associated with hypothyroidism in men patients with OSA after adjusting for potential confounding factors, and the optimal cutoff values to detect hypothyroidism in men with OSA was 10 score for ESS. In contrast, no significant association was found between ESS and hypothyroidism in women with OSA.

Extensive research has shown that OSA has an impact on the secretion of multiple hormones and is implicated in the development of many endocrine conditions, including obesity, diabetes mellitus, thyroid dysfunction, hypogonadism, osteoporosis, and so on (14). Hypothyroidism is a systemic metabolic syndrome characterized by decreased production/secretion of thyroid hormones or insufficient tissue utilization (12).The relationship between OSA and hypothyroidism has attracted more and more attention as some symptoms for both illnesses overlap. It was reported that repeated intermittent hypoxia and sleep fragmentation could induce impaired neuroendocrine regulation, which in turn lead to hypothalamic-pituitary-thyroid axis dysfunction (14). However, the specific pathophysiologic mechanism needs to be clarified.

We found that the prevalence of hypothyroidism in OSA population was 6.72%, and its rate in men OSA patients and women OSA patients was 5.12% and 10.38%, respectively. Some previous studies have reported the prevalence of hypothyroidism in OSA patients. Bahammam SA et al. reported that the prevalence of newly diagnosed clinical hypothyroidism in OSA patients was 0.4%, and the prevalence of newly diagnosed subclinical hypothyroidism was 11.1% (9). Petrone A et al. observed an 8% prevalence of subclinical hypothyroidism in the moderate to severe OSA patients (15). Bruyneel M et al. demonstrated that the prevalence of hypothyroidism in OSA patients was 1.4%, such that the prevalence of subclinical hypothyroidism was 7.5% (16). Ozcan KM et al. found that 1.97% clinical hypothyroidism and 10.8% subclinical hypothyroidism were diagnosed in patients with OSA, respectively (17). Mete T et al. declared that 4% OSA patients have clinical hypothyroidism and 4.7% OSA patients have subclinical hypothyroidism (7). This heterogeneity of prevalence between studies can be due to different definitions of hypothyroidism, difference in BMI, included participants, OSA severity, and so on. Although it is difficult to compare the prevalence of hypothyroidism in patients with OSA between different studies, a recent meta-analysis estimated the prevalence of clinical hypothyroidism in OSA patients was 8.12 ± 7.13% and that of subclinical hypothyroidism 11.07 ± 8.49% (10). In view of the above findings, it is declared that hypothyroidism should be paid enough attention in OSA patients although the epidemiological data is limited and somehow inconsistent.

The ESS, an eight-item questionnaire that asks whether the patient would doze off in various sedentary situations, is a well-known tool for assessing excessive daytime somnolence. On this scale, a score above 10 is suggestive of pathologic sleep-disordered breathing (18). Numerous papers have addressed the correlation between sleepiness and OSA and hypothyroidism. Misiolek M et al. reported the relationship between hypothyroidism and excessive daytime somnolence, and demonstrated that the hormonal stabilization in patients suffering from hypothyroidism could cause improvement in ESS (18). Resta O et al. also confirmed that ESS was significantly higher in the hypothyroid than the euthyroid subjects, and among the hypothyroid individuals, ESS was significantly higher in those with OSA than in those without OSA (19). Zhang M et al. in a meta-analysis including twelve studies and five case reports recruiting 192 hypothyroid OSA patients and 1423 euthyroid OSA patients suggested that ESS were significantly higher in the hypothyroid OSA patients when compared with euthyroid OSA patients (10). Moreover, Resta O et al. declared that OSA patients with subclinical hypothyroidism had a higher score of ESS as compared to the control group of OSA patients with normal thyroid function, while levothyroxine therapy could improve sleepiness (20). Consistent with this, in the present study, we found that OSA patients with hypothyroidism had a higher ESS score than OSA patients with normal thyroid function, although it not achieve statistical significance in women OSA patients. The results indicate that OSA and hypothyroidism all could increase sleepiness, and the overlap of the both disorders will aggravate it. Additionally, we found that ESS was the main difference between men OSA patients with hypothyroidism and women OSA patients with hypothyroidism, and ESS was positively associated with hypothyroidism in men patients with OSA after adjustment for potential confounding factors, and the optimal cutoff value for ESS to detect hypothyroidism in men patients with OSA was 10 score. ESS may have a potential role in identification and diagnosis of men OSA patients complicated with hypothyroidism.

It still remained controversial about whether to perform routine thyroid tests in patients with OSA. Some researchers have suggested routinely evaluating thyroid function in OSA patients, as they have a number of clinical features and symptoms in common, in order to avoid misdiagnosis, which may lead to failure on the treatment of continuous positive airway pressure and increase the risk of complications and mortality because of undiagnosed hypothyroidism (19). However, other researchers were skeptical of this view, because hypothyroidism was seen only in a small portion of patients with OSA (8). Even if there were some studies showing a relatively higher hypothyroidism, many biases were present in these studies (21). It has been proved that overt hypothyroidism and subclinical hypothyroidism were correlated with dyslipidemia, heart failure, atherosclerosis, hypertension, cardiovascular diseases, and metabolic disorders (22–24). Therefore, it is necessary to identify ones complicated with hypothyroidism among OSA patients as the overlap of the two disorders will increase the risk of cardiovascular disease and other complications, leading to increased overall morbidity and mortality. Our results showed a relatively higher prevalence of hypothyroidism in patients with OSA, especially in women OSA patients. In addition to, ESS was associated with hypothyroidism in men patients with OSA, and the prevalence of hypothyroidism in men OSA patients with ESS≥10 was significantly higher than that in OSA patients with ESS<10. So, we recommend evaluating thyroid function in patients with OSA, this balancing the clinical benefits with expenditure of medical resources and money in patient management.

The study has several limitations. Firstly, the association of ESS and hypothyroidism could not indicate causality due to the cross-sectional nature of this study. Secondly, the sample size of our study was relatively small, especially for women patients, which was mainly due to the relatively low prevalence of OSA in women compared with men. Thirdly, some potential confounding factors, such as dietary habits (especially iodine intake) and thyroid morphology, were not included in the multivariate regression analysis because these clinical data were missing. Finally, we performed thyroid function test only once in every patient with OSA in our study, not accounting for the possibility of transient hypothyroidism. Some research has reported that repeating the thyroid function measurements after 2–12 weeks may exclude some patients with transient hypothyroidism (9).

In conclusion, we found that a relatively higher prevalence of hypothyroidism in patients with OSA, especially in women OSA patients. The ESS was significantly and positively associated with hypothyroidism in men patients with OSA. In contrast, no significant association was observed between ESS and hypothyroidism in women patients with OSA. The results suggested a potential role of ESS in the identification and diagnosis of men OSA patients complicated with hypothyroidism. More researches and studies are needed to refine this evidence in the future.

## Data availability statement

The original contributions presented in the study are included in the article/supplementary material. Further inquiries can be directed to the corresponding authors.

## Ethics statement

The studies involving human participants were reviewed and approved by Ethics Committee of Tianjin Medical University General Hospital. Written informed consent for participation was not required for this study in accordance with the national legislation and the institutional requirements.

## Author contributions

All authors designed the study, collected data, reviewed the medical literatures, participated in the data analysis and interpretation, drafted and wrote the manuscript. All authors contributed to the article and approved the submitted version.

## Funding

This work was supported by Grants from the Natural Science Foundation of China (No. 81970084).

## Acknowledgments

We thank all participants from the sleep center of Department of Respiratory and Critical Care, Tianjin Medical University General Hospital.

## Conflict of interest

The authors declare that the research was conducted in the absence of any commercial or financial relationships that could be construed as a potential conflict of interest.

## Publisher’s note

All claims expressed in this article are solely those of the authors and do not necessarily represent those of their affiliated organizations, or those of the publisher, the editors and the reviewers. Any product that may be evaluated in this article, or claim that may be made by its manufacturer, is not guaranteed or endorsed by the publisher.

## References

[1] Senaratna CV Perret JL Lodge CJ Lowe AJ Campbell BE Matheson MC. Prevalence of obstructive sleep apnea in the general population: A systematic review. Sleep Med Rev (2017) 34:70–81. doi: 10.1016/j.smrv.2016.07.002 27568340

[2] Benjafield AV Ayas NT Eastwood PR Heinzer R Ip MSM Morrell MJ. Estimation of the global prevalence and burden of obstructive sleep apnoea: a literature-based analysis. Lancet Respir Med (2019) 7(8):687–98. doi: 10.1016/s2213-2600(19)30198-5 PMC7007763 31300334

[3] Louis JM Auckley D Sokol RJ Mercer BM. Maternal and neonatal morbidities associated with obstructive sleep apnea complicating pregnancy. Am J Obstet Gynecol (2010) 202(3):261.e1–5. doi: 10.1016/j.ajog.2009.10.867 20005507

[4] Cao J Wang Y Chen BY. Systemic damage and coping strategies of sleep apnea hypopnea syndrome. Chin J Lung Dis (Electronic Edition) (2014) 7(6):611–13. doi: 10.3877/cma.j.issn.1674-6902.2014.06.001

[5] Green ME Bernet V Cheung J. Thyroid dysfunction and sleep disorders. Front Endocrinol (Lausanne) (2021) 12:725829. doi: 10.3389/fendo.2021.725829 34504473 PMC8423342

[6] Akset M Poppe KG Kleynen P Bold I Bruyneel M. Endocrine disorders in obstructive sleep apnoea syndrome: A bidirectional relationship. Clin Endocrinol (Oxf) (2022) 1–11. doi: 10.1111/cen.14685 35182448

[7] Mete T Yalcin Y Berker D Ciftci B Guven Firat S Topaloglu O. Relationship between obstructive sleep apnea syndrome and thyroid diseases. Endocrine (2013) 44(3):723–8. doi: 10.1007/s12020-013-9927-9 23564558

[8] Takeuchi S Kitamura T Ohbuchi T Koizumi H Takahashi R Hohchi N. Relationship between sleep apnea and thyroid function. Sleep Breath (2015) 19(1):85–9. doi: 10.1007/s11325-014-0966-0 24622960

[9] Bahammam SA Sharif MM Jammah AA Bahammam AS. Prevalence of thyroid disease in patients with obstructive sleep apnea. Respir Med (2011) 105(11):1755–60. doi: 10.1016/j.rmed.2011.07.007 21820299

[10] Zhang M Zhang W Tan J Zhao M Zhang Q Lei P. Role of hypothyroidism in obstructive sleep apnea: a meta-analysis. Curr Med Res Opin (2016) 32(6):1059–64. doi: 10.1185/03007995.2016.1157461 26907534

[11] Berry RB Budhiraja R Gottlieb DJ Gozal D Iber C Kapur VK. Rules for scoring respiratory events in sleep: update of the 2007 AASM manual for the scoring of sleep and associated events. deliberations of the sleep apnea definitions task force of the American academy of sleep medicine. J Clin Sleep Med (2012) 8(5):597–619. doi: 10.5664/jcsm.2172 23066376 PMC3459210

[12] Endocrinology branch of Chinese Medical Association. Guidelines on diagnosis and treatment of hypothyroidism in adults in China. Chin J Endocrinol Metab (2017) 33(2):167–80. doi: 10.3760/cma.j.issn.1000-6699.2017.02.018

[13] Chinese Medical Association. Guideline for primary care of hyperthyroidism (2019). Chin J Gen Pract (2019) 18(12):1118–28. doi: 10.3760/cma.j.issn.1671-7368.2019.12.002

[14] Lavrentaki A Ali A Cooper BG Tahrani AA. MECHANISMS OF ENDOCRINOLOGY: Mechanisms of disease: the endocrinology of obstructive sleep apnoea. Eur J Endocrinol (2019) 180(3):R91–r125. doi: 10.1530/eje-18-0411 30540561

[15] Petrone A Mormile F Bruni G Quartieri M Bonsignore MR Marrone O. Abnormal thyroid hormones and non-thyroidal illness syndrome in obstructive sleep apnea, and effects of CPAP treatment. Sleep Med (2016) 23:21–5. doi: 10.1016/j.sleep.2016.07.002 27692273

[16] Bruyneel M Veltri F Poppe K. Prevalence of newly established thyroid disorders in patients with moderate-to-severe obstructive sleep apnea syndrome. Sleep Breath (2019) 23(2):567–73. doi: 10.1007/s11325-018-1746-z 30368659

[17] Ozcan KM Selcuk A Ozcan I Ozdas T Ozdogan F Acar M. Incidence of hypothyroidism and its correlation with polysomnography findings in obstructive sleep apnea. Eur Arch Otorhinolaryngol (2014) 271(11):2937–41. doi: 10.1007/s00405-014-2962-1 24609648

[18] Misiolek M Marek B Namyslowski G Scierski W Zwirska-Korczala K Kazmierczak-Zagorska Z. Sleep apnea syndrome and snoring in patients with hypothyroidism with relation to overweight. J Physiol Pharmacol (2007) 58 Suppl 1:77–85. 17443029

[19] Resta O Pannacciulli N Di Gioia G Stefàno A Barbaro MP De Pergola G. High prevalence of previously unknown subclinical hypothyroidism in obese patients referred to a sleep clinic for sleep disordered breathing. Nutr Metab Cardiovasc Dis (2004) 14(5):248–53. doi: 10.1016/s0939-4753(04)80051-6 15673058

[20] Resta O Carratù P Carpagnano GE Maniscalco M Di Gioia G Lacedonia D. Influence of subclinical hypothyroidism and T4 treatment on the prevalence and severity of obstructive sleep apnoea syndrome (OSAS). J Endocrinol Invest (2005) 28(10):893–8. doi: 10.1007/bf03345320 16419491

[21] Holley AB. Should you screen all your sleep apnea patients for thyroid disease? Sleep Breath (2015) 19(1):21–2. doi: 10.1007/s11325-014-0998-5 24840213

[22] Asvold BO Bjøro T Nilsen TI Vatten LJ. Association between blood pressure and serum thyroid-stimulating hormone concentration within the reference range: a population-based study. J Clin Endocrinol Metab (2007) 92(3):841–5. doi: 10.1210/jc.2006-2208 17200168

[23] Ochs N Auer R Bauer DC Nanchen D Gussekloo J Cornuz J. Meta-analysis: subclinical thyroid dysfunction and the risk for coronary heart disease and mortality. Ann Intern Med (2008) 148(11):832–45. doi: 10.7326/0003-4819-148-11-200806030-00225 18490668

[24] Cooper DS Biondi B. Subclinical thyroid disease. Lancet (2012) 379(9821):1142–54. doi: 10.1016/s0140-6736(11)60276-6 22273398

